# NDC80/HEC1 promotes macrophage polarization and predicts glioma prognosis via single‐cell RNA‐seq and in vitro experiment

**DOI:** 10.1111/cns.14850

**Published:** 2024-07-17

**Authors:** Weijie Ye, Xisong Liang, Ge Chen, Qiao Chen, Hao Zhang, Nan Zhang, Yuanfei Huang, Quan Cheng, Xiaoping Chen

**Affiliations:** ^1^ Department of Clinical Pharmacology, Xiangya Hospital Central South University Changsha China; ^2^ Hunan Key Laboratory of Pharmacogenetics, Institute of Clinical Pharmacology Central South University Changsha China; ^3^ Department of Neurosurgery, Xiangya Hospital Central South University Changsha China; ^4^ Department of Neurosurgery, The Second Affiliated Hospital Chongqing Medical University Chongqing China; ^5^ College of Life Science and Technology Huazhong University of Science and Technology Wuhan Hubei China

**Keywords:** glioma, HEC1, prognosis, scRNA‐seq, tumor microenvironment

## Abstract

**Introduction:**

Glioma is the most frequent and lethal form of primary brain tumor. The molecular mechanism of oncogenesis and progression of glioma still remains unclear, rendering the therapeutic effect of conventional radiotherapy, chemotherapy, and surgical resection insufficient. In this study, we sought to explore the function of HEC1 (highly expressed in cancer 1) in glioma; a component of the NDC80 complex in glioma is crucial in the regulation of kinetochore.

**Methods:**

Bulk RNA and scRNA‐seq analyses were used to infer HEC1 function, and in vitro experiments validated its function.

**Results:**

HEC1 overexpression was observed in glioma and was indicative of poor prognosis and malignant clinical features, which was confirmed in human glioma tissues. High HEC1 expression was correlated with more active cell cycle, DNA‐associated activities, and the formation of immunosuppressive tumor microenvironment, including interaction with immune cells, and correlated strongly with infiltrating immune cells and enhanced expression of immune checkpoints. In vitro experiments and RNA‐seq further confirmed the role of HEC1 in promoting cell proliferation, and the expression of DNA replication and repair pathways in glioma. Coculture assay confirmed that HEC1 promotes microglial migration and the transformation of M1 phenotype macrophage to M2 phenotype.

**Conclusion:**

Altogether, these findings demonstrate that HEC1 may be a potential prognostic marker and an immunotherapeutic target in glioma.

## Graphical abstract

In summary, this study has revealed the role of NDC80/HEC1 in the process of glioma development and in the tumor microenvironment of glioma. The immunosuppressive property and role of HEC1 make it attractive to be used as novel prognostic biomarkers or effective therapeutic targets. Immunofluorescence of glioma tissue microarrays showed positive correlation of HEC1 expression with the malignancy degrees of glioma and a lower probability of patients with high HEC1 expression. In particular, HEC1 promotes development of glioma through the regulation of cell proliferation, cell cycle, DNA repair, and TME formation, possibly through transcriptional activation of E2F8. Moreover, tumor cells exhibiting HEC1 expression could shape the TME by interacting with macrophage and cancer‐associated fibroblasts. Coculture confirmed that glioma cells devied‐HEC1 promote macrophage migration and transform into M2. Our results provide further insight into the application of HEC1 in glioma (by Figdraw).

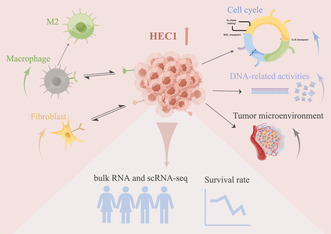

## 1 INTRODUCTION

Glioma is the most common primary tumor of the central nervous system. It accounts for 48.3% of all brain malignancies, with only 6.8% 5‐year relative survival rate.^1^ The current treatment of glioma mostly includes effective and safe treatments such as surgery, radiation, and temozolomide chemotherapy. Nonetheless, these treatment modalities are extremely difficult because of the complexities in complete surgical resection and chemotherapy.^2^, ^3^ Despite various emerging therapeutic strategies such as molecular targeted therapy, immunotherapy, and electric field therapy, little progress has been made in the area of glioma. Thus, further insights are needed into the molecular mechanisms underlying glioma progression to develop novel therapeutic strategies for the malignancy.

Glioma cells are surrounded by a highly dynamic and complex tumor microenvironment (TME) comprising various cell types and are highly relevant for an effective immunotherapy.^4^ Suppressive immune cells such as tumor‐associated macrophages and immunosuppressive cytokines are closely associated with the formation of immunosuppressive TME in glioma.^5^ Immune checkpoint molecules are also important for mediating immunosuppression in the TME. Therapies involving immune checkpoint blockade are recognized as a promising approach to combat cancers.^6^

The centromere, an evolutionarily conserved protein complex with multiple subunits, plays a central role in chromosome segregation and genome isolation. The centromere is frequently dysregulated in cancer. Over the past 30 years, many studies have been conducted to examine the structure and function of the centromere. It is now evident that the NDC80 kinetochore complex, MIS12 complex (*Mis12*, *Nnf1*, *Nsl1*, and *Dsn1*), and KNL1 complex (*Kinetochore scaffold 1* and *ZW10 interacting kinetochore protein*, *Spc105*, and *Ydr532* yeast) co‐participate in spindle physical examination point signal and mitotic spindle assembly.^7^, ^8^ The NDC80 complex comprises four subunits, *Hec1*, *Nuf2*, *Spc24*, and *Spc25*, that play vital roles in centromere and microtubule attachment.^9^ Nonetheless, little is known about how the abundance of individual centromeric subunits is regulated in specialized cellular environments such as meiosis.^10^

Highly expressed in cancer 1 (*HEC1*), also called NDC80 kinetochore complex component (*NDC80*), is one of the major functional component of the centromere.^11^ *Hec1* is phosphorylated by Monopolar spindle1 (*Mps1*) to activate the spindle assembly checkpoint (SAC) and cause mitotic arrest.^12^ In addition, NIMA related kinase 2 (*Nek2*) mediated phosphorylation of *Hec1* may affect chromosomes separation.^13^ Evidence suggests increased expression of HEC1 in several cancers, including hepatocellular carcinoma (HCC),^14^ osteosarcoma,^15^ and breast cancer.^16^ HEC1 also plays crucial roles in cancer development and progression.^17^ For instance, HEC1 contributes to colon cell proliferation and metastasis. Several studies have also shown enhanced expression of SAC regulators (as *Mps1*, *Nek2*, *Mitotic arrest‐deficient 1*, *Mitotic arrest‐deficient 2*) in cancer with HEC1 overexpression.^18^ Thus, HEC1 may possibly interact with these proteins that regulate SAC function.^18^ In HCC, HEC1 correlates positively with immune infiltration cells, including regulatory T cells, macrophages, neutrophils, and dendritic cells.^19^ HEC1 also played a role in programmed death ligand 1 (PD‐L1) inhibitor treatment of patients with HCC.^20^ However, whether HEC1 could influence the tumor immune microenvironment is not known. A few studies have also examined the biological functions of HEC1 in glioma and observed overexpression of HEC1 in glioma cells and associated with their proliferation and invasion.^21^ However, the molecular mechanism of HEC1 in regulating glioma remains to be elucidated.

Herein, we analyzed the potential function of HEC1 in glioma and the relationship between HEC1 and macrophage in the TME through bioinformatics analysis. Following this, in vitro cellular experiments were performed to verify the function of HEC1. Finally, the relationship between HEC1 expression and glioma prognosis was analyzed by multiplexed immunofluorescence of human glioma tissue. Our findings reveal a novel mechanism that HEC1 modulates tumor cell proliferation, and that macrophage migration and polarization promote glioma growth. HEC1 could possibly serve as a potential target for glioma.

## 2 MATERIALS AND METHODS

### 2.1 Cell culture and transfection

U251, U87, and HMC3 cells were obtained from the National Infrastructure of Cell Line Resource, China. U251 and U87 cells were grown in high or low glucose Dulbecco's modified Eagle's medium (DMEM, Gibco) with 10% fetal bovine serum (FBS, Gibco), respectively. HMC3 cells were grown in Roswell Park Memorial Institute (RPMI) 1640 medium (Gibco) with 10% FBS. *SiRNA* transfection was performed using RNAiMAX (Invitrogen) according to the protocols. HEC1 siRNA1: (5′‐CCACUUAAUGACAAAGCAUTT‐3′, 5′‐AUGCUUUGUCAUUAAGUGGTT‐3′); HEC1 siRNA2: (5′‐GGAGCAGAUUGCUAAAGUUTT‐3′, 5′‐AACUUUAGCAAUCUGCUCCTT‐3′).

### 2.2 Proliferation assay

The proliferation of glioma cell was detected through clone formation, CCK8 assay, and EdU assay. For clone formation, 10^3^ cells were seeded in 6‐well plate. After 2 weeks, the medium was changed, and after 3 weeks, the cells were stained with crystal violet (Beyotime Biotechnologhy) and observed under a microscope (Aglient Biotek Cytation5, Biotek). For the CCK8 assay, 2 × 10^3^ cells were seeded into a 96‐well plate, and 10% CCK8 (bimake.cn) was added to the cells after 0, 24, 48, and 96 h. After 1 h of incubating with CCK8, the cell culture plate was read using a microplate reader (Varioskan LUX, Thermo) at a wavelength of 490 nm. For the EdU assay, 2 × 10^3^ cells were seeded in a 96‐well plate for 24 h and incubated with 10 nM EdU (Beyotime Biotechnolohy) for 1 h, following the instructions and observed under a fluorescence microscope (Aglient Biotek Cytation5, Biotek).

### 2.3 Real‐time polymerase chain reaction (Real‐time PCR)

RNA was extracted using RNAiso Plus reagent (Takara) and transcribed into cDNA using PrimeSript™ RT reagent kit (Takara). Real‐time PCR was performed using TB Green (Takara). The mean cycle threshold (Ct) values were used to calculate fold changes. Relative gene expression was calculated employing the 2^−△△CT^ method. The primer sequences for β‐actin were as follows: 5′‐CGGGAAATCGTGCGTGAC‐3′ (Forward), 5′‐TGGAAGGTGGACAGCGAGG‐3′ (Reverse); HEC1: 5′‐CCAGAGGCAAAGAAGCGATTG‐3′ (Forward), 5′‐GCACCAGCCTCGGGATTAAA‐3′ (Reverse).

### 2.4 Analysis of protein expression

Total proteins were extracted using the Protein Extraction Kit (Beyotime Biotechnology) following the manufacturer's protocol. Samples were prepared following the instructions (Revision 1.0), and protein expression was determined using JESS (ProteinSimple Instruments, bio‐techne). Western blot was performed using Abcam Western blot methods.

### 2.5 HMC3 and glioma cell coculture for migration study

The migration of microglia cells (HMC3) was detected by transwell assay. The glioma cells (U251/U87) were seeded in a 6‐well plate at density of 4 × 10^4^ cells/well. The cells were transfected with HEC1 siRNA or control RNA, the continuously cultured for another 24 h. The glioma cells were then digested with trypsin (Gibco) and resuspended at concentration of 2 × 10^4^ cells/mL and 1 mL cells were added to the lower chamber. HMC3 cells were also digested with trypsin and resuspended at 2 × 10^5^ cells/mL and 500 μL cells were added to the upper chamber. Cells were cocultured together for 48 h and washed thrice with phosphate buffered saline (PBS). For crystal violet staining, cells were fixed with 4% paraformaldehyde (Beyotime Biotechnology) and stained with 0.1% crystal violet (Beyotime Biotechnology) for 15 min and dried overnight. The cells were then viewed under a microscope (DSZ2000X, Beijing Zhongxian Hengye instrument).

### 2.6 HMC3 and glioma cell coculture for flow cytometry assay

The glioma cells (U251/U87) were seeded at a 6‐well plate at 4 × 10^4^ cells/well. The cells were transfected with HEC1 siRNA or control RNA, continuously cultured for another 24 h, and then digested with trypsin and resuspended at concentration of 5 × 10^5^ cells/mL and 100 μL cells were added to the upper chamber. HMC3 cells were digested with trypsin and resuspended in the medium, and 5 × 10^4^ cells were added to the lower chamber. Cells were cocultured for 48 h. HMC3 cells in the lower chamber were trypsinized, and the cell precipitate was obtained by centrifugation. The cells were washed with PBS thrice, and CD68 antibody (eBioscience), CD86 antibody (eBioscience), or CD163 antibody (eBioscience) was added and incubated at 4°C for 30 min, in dark. After washing with PBS, 200 μL of PBS was added to suspend the cells and detected by a flow cytometer (A00‐1‐1102, Beckman).

### 2.7 Bioinformatics analysis

Kaplan–Meier (K‐M) analysis and log‐rank test were conducted to analyze overall survival (OS), progression free survival (PFI), and disease specific survival (DSS). The receiver operating characteristic (ROC) curve and area under the curve (AUC) were employed to evaluate the predictive discrimination of different classification approaches, including survival rates, glioma subtype, and isocitrate dehydrogenase 1 (IDH1) genotype. Hazard ratios (HRs) with 95% confidence intervals (CI) were estimated with log‐rank *p* values.

The Estimation of STromal and Immune cells in MAlignant Tumor tissues using Expression data (ESTIMATE) algorithm was used to assess Stromal score, Immune Score and ESTIMATE Score, as previously reported.^22^ Next, Gene Set Enrichment Analysis (GSEA) and Gene Set Variation Analysis (GSVA) were performed following previous studies.^23^ The Immunedeconv R package was used to assess immune infiltration, which integrates six immune infiltration algorithms, including xCell and CIBERSORT.

### 2.8 Single‐cell RNA sequencing of the public datasets

The Gene Expression Omnibus (GEO) dataset (GSE138794, GSE84465, GSE131928) and a data set (scp50) in single‐cell portal (SCP) as reference were merged; the batch effects were removed and integrated with Harmony.^24^ Quality control, standardization, normalization, principal component analysis, and Uniform Manifold Approximation and Projection were performed according to standard Seurat procedures. Tumor cells were identified using the copy number variations (CNV) algorithm, and high and low expression groups were determined employing Scissor. Tumor cells were classified into subtypes astrocyte (AC), mesenchymal (MES), neural progenitor (NPC), and oligodendrocyte‐progenitor (OPC), as previously defined by Neftel et al.^25^ A pseudotemporal analysis of tumor cells was performed using Monocle,^26^ which ranked trajectories based on the first 2000 key genes and clustered the distribution of tumor cells according to their clusters and subtypes.

The differentiation status and tumor cell order were examined using CytoTRACE.^27^ Then, differential analysis of the cells of the branch cluster of key nodes was performed, and the differentially expressed genes were annotated using Gene Ontology (GO) and Kyoto Encyclopedia of Genes and Genomes (KEGG). The heterogeneous phenotypes of the different groups caused by the diversity in transcriptional regulation were examined using pySCENIC to obtain differentially expressed regulators.

To infer cell cycle position at high resolution from scRNA‐seq data, Tricycle software package was employed using key features of cell cycle biology, features of principal component mathematics periodic function, and transfer learning. The estimate cycle position function was employed to determine cell cycle position, infer cell cycle, and evaluate performance, followed by the use of the plot_ccposition_den function to calculate the kernel density of θ from the von Mises distribution, with the output being a Cartesian plot.

The relative abundance of metabolites was inferred using the Python‐based software package MEBOCOST,^28^ based on the gene expression of metabolic reaction enzymes. All cell‐to‐cell interactions based on coimmunosuppressive ligands and receptors were confirmed using CellphoneDB. The multilayer network was analyzed by using scRNA‐seq‐based cell–cell communication inference tools.^29^, ^30^

### 2.9 Tissue chip immunofluorescence

A tumor tissue chip (catalog No. BraSur2201) contains glioma tissue was provided by the Life Sciences Ethics Committee of Changsha Yaxiang Biotechnology Co., LTD with ethical approval. The tumor tissue chip was routinely deparaffinized, hydrated, and high‐pressure antigen repaired. The chip was then blocked and incubated with HEC1 antibody (1:100, proteintech), Glial fibrillary acidic protein (GFPA) antibody (1:200, proteintech), CD68 antibody (1:100, abcam), and CD163 antibody (1:100, abcam) overnight at 4°C. After incubation with the second antibodies, the chip was labeled with Tyramide Signal Amplification fluorescent dye (Thermo). Nuclei were counterstained with *DAPI* stain (Thermo).

### 2.10 Statistical analysis

Samples were grouped based on the median of the HEC1 expression. The Shapiro–Wilk test and Kolmogorov–Smirnov test were used to assess data distribution. Student's *t*‐test and one‐way analysis of variance (ANOVA) were used to compare normally distributed variables between the two groups and multiple groups, respectively. The Wilcoxon test and Kruskal–Wallis test were utilized to compare the non‐normally distributed data between the two groups and multiple groups, respectively. Data were analyzed and plotted using R (version 4.2), and statistically significant values were represented by GraphPad Prism (version 8.0) and *p* < 0.05.

## 3 RESULTS

### 3.1 The association of high HEC1 expression with poor prognosis and clinical features and was involved in cell cycle, DNA repair, and tumor microenvironment in glioma

**FIGURE 1:**
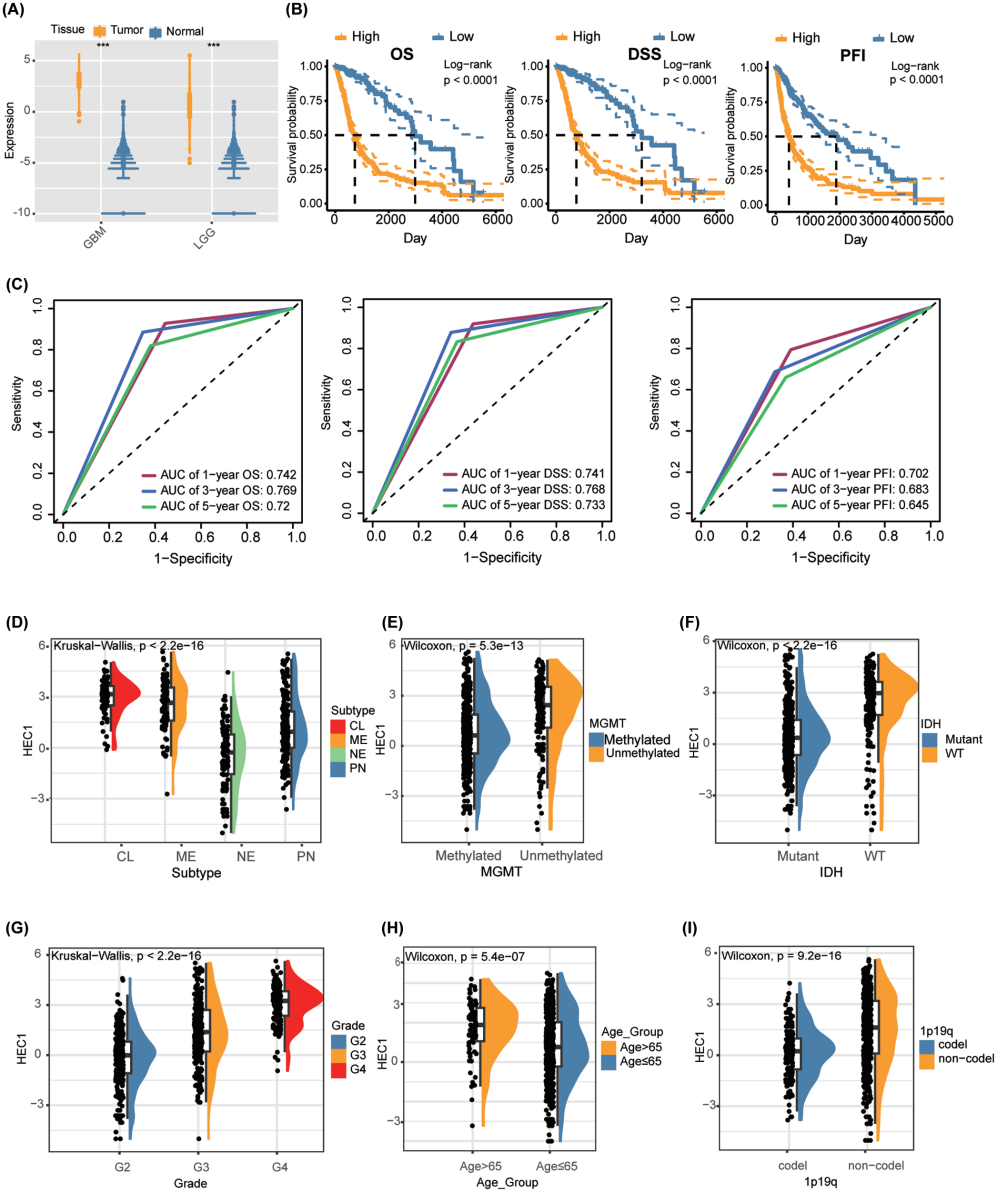
*HEC1* expression levels and the prognostic value in glioma. (A) HEC1 mRNA expression in glioma samples compared to that of normal brain samples. (B) Survival curves of overall survival (OS), disease specific survival (DSS), and progression free survival (PFI) between high and low HEC1 expressions in patients. (C) Nomogram to predict OS, DSS, and PFI according to HEC1 expression. (D–I) The expression of HEC1 in different glioma subtypes (D), MGMT methylation status (E), isocitrate dehydrogenase 1 (IDH) genotypes (F), the World Health Organization grade (G), age (H), and 1p/19q co‐deletion status (I).

We first compared the difference in HEC1 expression between glioma and control tissues using public data from The cancer genome atlas (TCGA) and Genotype‐Tissue Expression and found significantly higher HEC1 expression in glioma tissues compared with controls (Figure 1A). Based on median HEC1 expression, the samples were categorized into high (*n* = 334) and low HEC1 expression (*n* = 337) groups; the median values of expression of HEC1 were 1.1117. Kaplan–Meier (K‐M) survival analysis was then performed. The high HEC1 expression group had lower overall survival (OS), progression free survival (PFI), and disease specific survival (DSS) than the low HEC1 expression group (Figure 1B). Receiver operating characteristic (ROC) curve analysis evaluated the accuracy of the high HEC1 expression in predicting the disease prognosis. The 1‐, 3‐, and 5‐year OS, DFS, and PFS were all well predicted by this subgroup (Figure 1C). Analysis of the association between HEC1 expression and various clinical features revealed higher HEC1 expression levels in patients with classical and subtypes, O6‐methylguanine‐DNA methyltransferase (MGMT) unmethylated, isocitrate dehydrogenase (IDH) wild‐type, high World Health Organization grade (WHO) grade, over 65 years old, or 1p/19q noncodeletion (Figure 1D–I).

To investigate the possible difference in biological pathways the high and the low HEC1 expression groups, we performed differential gene analysis (Figure S1A). Then, enrichment analysis of the different genes using Gene Set Enrichment Analysis (GSEA) and Gene Set Variation Analysis (GSVA) showed that the high HEC1 expression group was enriched in the biological processes related to antigen presentation, DNA repair, cell cycle, extracellular matrix (ECM), and cytokine receptor ligand interaction (Figure S1B,C). Consistently, signaling pathways related to DNA replication checkpoint, DNA double‐strand repair, mismatch repair, and homologous recombination repair, cytokines, and ECM were higher in the high HEC1 expression group (Figure S1D,E). These findings suggest that HEC1 is a potential oncogene of glioma. Higher HEC1 levels are associated with poor prognosis and malignant clinical features, which may function through regulation cell cycle, DNA repair, and TME.

### 3.2 The association of high HEC1 expression in glioma with tumor‐promoting tumor characteristics, including strong immune cells infiltration, and increased immune checkpoint expression

**FIGURE 2:**
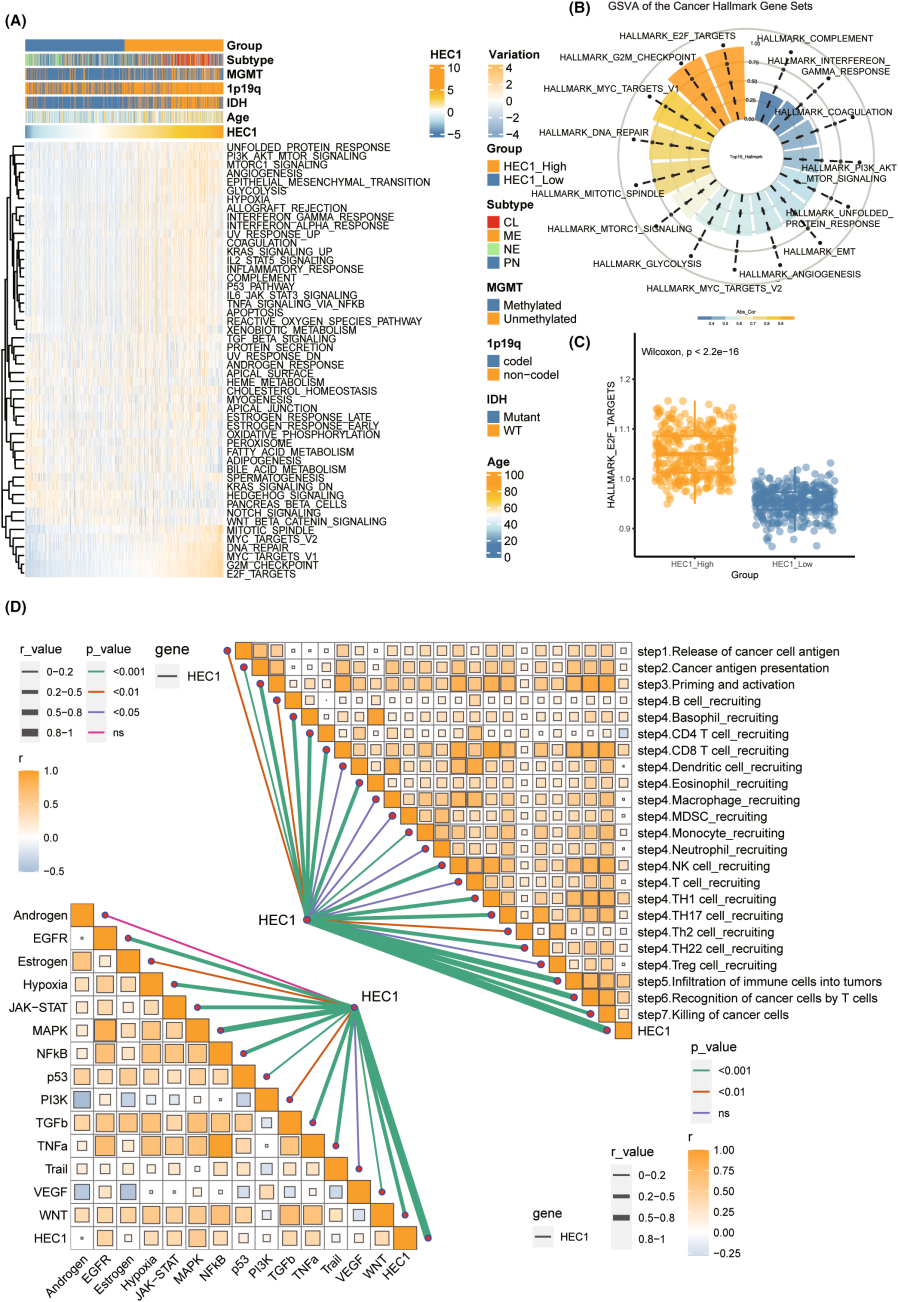
Comparison of HEC1 function and immune pathways. (A) Heatmap showing the correlation between HEC1 expression levels and hallmark signatures according to single‐strand Gene Set Enrichment Analysis (ssGSEA). (B) Doughnut diagram showing Spearman correlations between HEC1 expression levels and hallmark enrichment. (C) Comparison of the E2F target gene enrichment between high and low HEC1 expressions. (D) Spearman correlations between HEC1 expression and the step cycle of the anti‐tumor immunesystem (right) or the classical tumor‐associated PROGENy pathways (left) in the butterfly bar plot.

We found enhanced levels of several tumor features, including E2F targets, G2M checkpoints, and DNA repair, with the increase in expression of HEC1 (Figure 2A). Then, the correlation of HEC1 expression with tumor characteristics was determined using by Spearman correlation analysis, and the top 15 characteristics exhibiting the highest correlation coefficient were identified. The correlation between E2F target characteristics and HEC1 expression was >0.9 (Figure 2B). Compared with the low HEC1 expression group, the high HEC1 expression group had higher values for E2F target characteristics (Figure 2C). We also evaluated the association of HEC1 levels with the classical tumor pathways and anti‐tumor immune circulatory pathways and found positive correlation of HEC1 levels with all classical tumor pathways except androgen, as well as numerous immune activity pathways (Figure 2D).

To understand the role of HEC1 in tumor immunity better, we evaluated immune infiltration by ESTIMATE algorithm. The high HEC1 expression group had higher ESTIMATE score, immunity score, stroma score, and lower tumor purity (Figure S2A). Furthermore, macrophages and cancer‐associated fibroblasts (CAFs) infiltration were significantly higher in the high HEC1 expression group (Figure S2B–D). Most immune checkpoints, including *PD‐L1* and *Programmed cell death 1* (*PDCD1*), involved in immune escape of tumor cells, were also highly expressed in the high HEC1 expression group (Figure S3A–C). All these results suggested the possible involvement of HEC1 in tumorigenesis and tumor immunity, especially in the formation of tumor immune microenvironment.

### 3.3 Tumor cells with high HEC1 expression were characterized by high E2F target levels, DNA repair, and cell cycle

**FIGURE 3:**
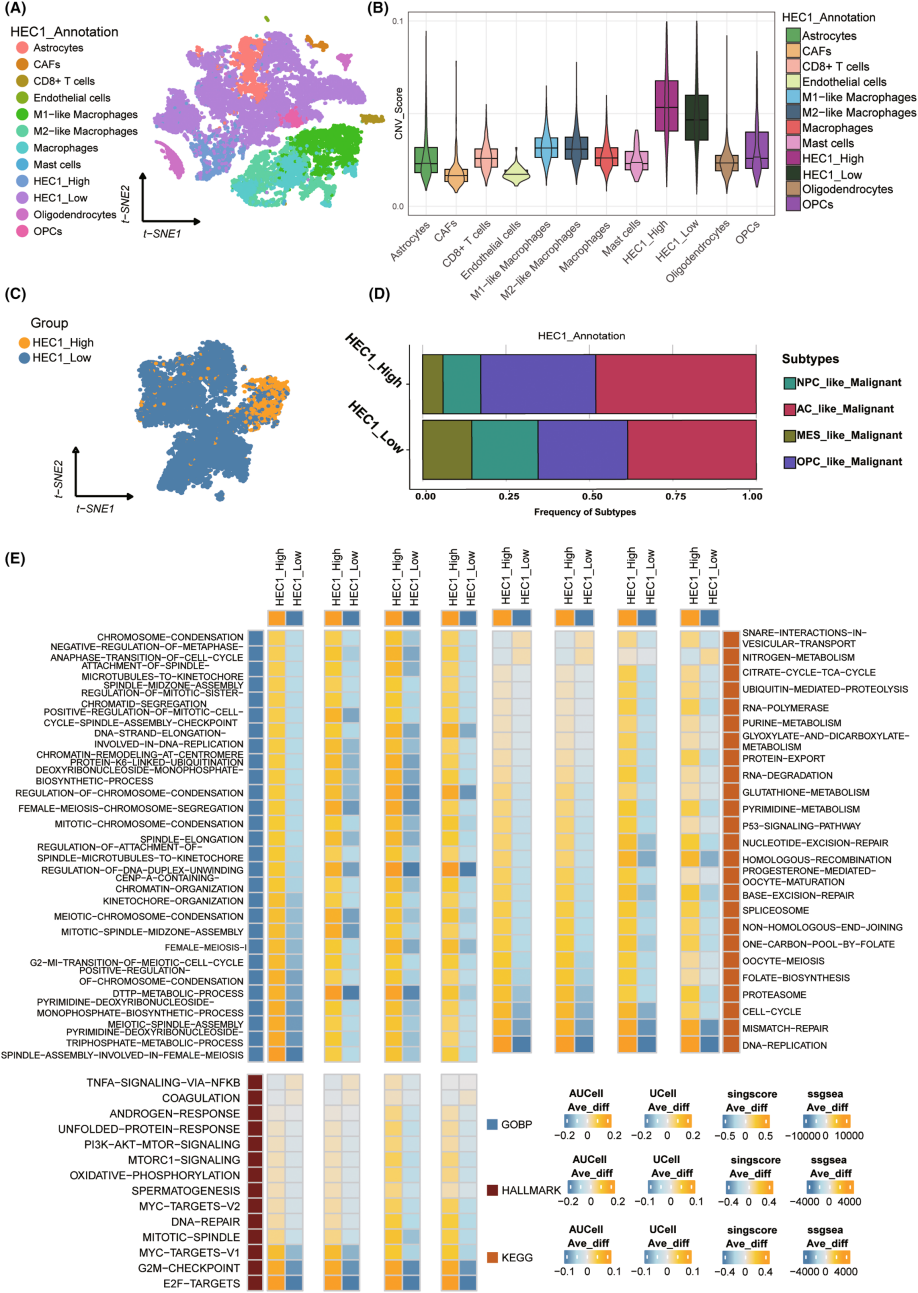
Expression and functional analysis of HEC1 based on single‐cell data sets. (A) t‐distributed stochastic neighbor embedding (tSNE) plot of single‐cell transcriptomes. (B) The copy number score of all cell populations in the boxplot. (C) Tumor cells were grouped according to the expression of HEC1 in tSNE. (D) Bar chart showing the different nodes of glioblastoma subtypes between groups with high or low HEC1 levels. (E) Heatmap showing differential enrichment in Gene Ontology, Kyoto Encyclopedia of Genes and Genomes and HALLMARK between groups with high or low HEC1 levels.

Next, the role of HEC1 in glioma was validated at the single‐cell level. First, a multiple cell population was revealed by clustering the combined datasets using uniform multiplicity approximation and projection (Figure 3A). Cells were identified by copy number variations (CNV) score, with the CNV score of tumor cells being higher than that of other cells (Figure 3B). A total of 14,390 tumor cells were classified; there were 12,720 cells with low and 1670 cells with high HEC1 expression, and the median value of HEC1 expression was 0 (Figure 3C). The expression of HEC1 was >0 in the high expression group and 0 in the low expression group. The CNV score of the high HEC1 expression group was higher than that of the low HEC1 expression group. The t‐distributed stochastic neighbor embedding (tSNE) diagram and the Violin diagram showed the HEC1 expression level in all cell clusters, and we noticed particularly higher HEC1 expression in the HEC1_High tumor cells (Figure S4A,B). The Figure S4C shows the proportions of HEC1_High cells and HEC1_Low cells of the individual samples in Figure S4A above. Sample MGH143 contributed the most to the HEC1_High cells (Figure S4D), and the fold change of M2 macrophages to M1 macrophages was greater in these samples (Figure S4E). In addition, comparison of the proportions of the four glioblastoma (GBM) subtypes in the two groups found a higher proportion of astrocyte (AC) and oligodendrocyte‐progenitor (OPC) subtypes in the high HEC1 expression group (Figure 3D). The differences in pathway enrichment were evaluated using four subtypes. GO showed that the group with high HEC1 expression had more biological processes related to DNA replication and metabolism, whereas KEGG and HALLMARK showed that the high HEC1 expression group had more biological activities related to DNA activity, such as high E2F targets, the G2M checkpoint, and DNA repair (Figure 3E).

To elucidate the mechanism of HEC1 function in glioma, differential and enriched transcription factor analysis was performed. First, the transcription factors were clustered into 11 modules (Figure S5A), among which, modules M3 and M10 were significantly enriched in the high HEC1 expression group (Figure S5B,C). Then, differential expression analysis of all transcription factors (Figure S5D) was performed and crossed the highly expressed transcription factors with the highly enriched transcription factor modules in the high HEC1 expression group. The transcription regulator corresponding to E2F8 exhibited the highest specificity (Figure S5E,F). The distribution of HEC1 and E2F8 exhibited similar expression profiles (Figure S5G), suggesting their significant relevance. As expected, tumor cells with high E2F8 expression were associated with biological processes of DNA activity and were enriched in cell cycle and pathways related to DNA repair (Figure S5H). These results suggested that the tumor cells expressing high levels of HEC1 had higher malignant biological phenotype and that E2F8 may be involved in HEC1‐mediated DNA replication and repair.

### 3.4 The tumor cells with different expression of HEC1 exhibit heterogeneity of cell cycle and interaction

**FIGURE 4:**
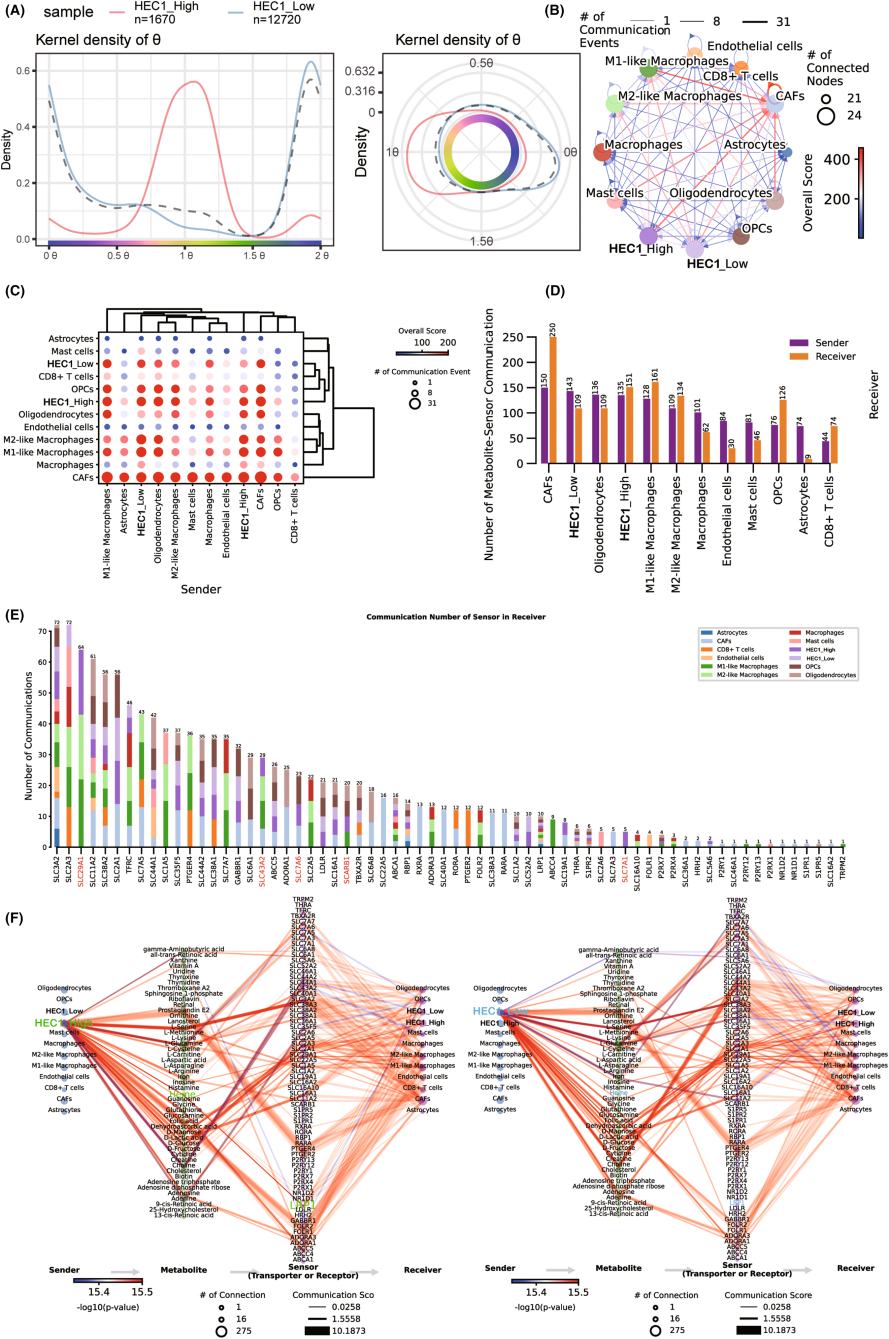
The analysis of interaction between cell cycle and metabolism of tumor cells expressing high or low HEC1 levels. (A) The cell cycle distributions of tumor cells with high (left) or low (right) expression levels of HEC1. (B) The overall interactions between all cell populations. (C) The number of different cell populations involved in metabolic interactions as sender and receiver. (D) The levels of interaction of different cell populations involved in cellular metabolic interactions as sender and receiver. (E) The proportions of cell populations participating in metabolic interactions mediated by each gene. (F) The pathways for metabolic interaction of tumor cells with high (left) and low (right) expressing HEC1, as transmitter, receiver, and receptor/transporter interaction with other cell populations.

The heterogeneity of cell cycle and metabolism were evaluated by Tricycle and MEBOCOST. Most of the tumor cells in the high HEC1 and low expression groups were in S‐M phase and G0/G1 phase, respectively (Figure 4A), indicating that the tumor cells in the high HEC1 expression group had strong proliferation activity. The tumor cells in the high HEC1 expression group had enhanced metabolic interaction with macrophage and CAFs (Figure 4B,C). The transmitter–receiver metabolic interactions of all cells were compared and showed that tumor cells in the high HEC1 expression group had higher receiver metabolic activity than those in the low HEC1 expression group (Figure 4D). In addition, when metabolic interactions were analyzed, the group with high HEC1 expression exhibited more characteristic metabolic interactions mediated by SLC29A1, SLC43A2, SLC7A6, and SCARB1 (Figure 4E). When tumor cells acted as transmitters, the high HEC1 expression group could specifically target other cells, including macrophage and CAFs, through the heme‐LRP1 receptor (Figure 4F).

To clarify the interaction between the high HEC1 and the low HEC1 expressing groups further, we analyzed the cell interactions mediated by receptor and ligand pairs in the context of immunosuppression. The interaction of tumor cells in the high HEC1 expression group was more with CAFs and macrophage; these cells interacted more with CAFs through *signal regulatory protein alpha* (*SIRPA*)‐*CD47* (Figure S6A), *adiponectin* (*ADIPOQ*)‐*adiponectin receptor 2* (*ADIPOR2*), *Wnt family member 5A* (*WNT5A*)‐*melanoma cell adhesion molecule* (*MCAM*), *interleukin 11* (*IL11*)‐(*IL11 subunit alpha* (*IL11RA*) + *IL 6 cytokine family signal transducer* (*IL6ST*)), *heparin binding EGF like growth factor* (*HBEGF*)‐*epidermal growth factor receptor* (*EGFR*), and *vascular endothelial growth factor A* (*VEGFA*)—*fms related receptor tyrosine kinase 1*(*VEGFR1*) (Figure S6B–F), while in macrophage, they were mediated through *HBEGF‐EGFR* (Figure S6E). Moreover, CD8^+^ T cell interacted with tumor cells and CAFs in the high HEC1 expression group by *granzyme A* (*GZMA*)—*par‐3 family cell polarity regulator* (*PARD3*) (Figure S6G). These findings suggest that tumor cells with high HEC1 expression exhibit heterogeneity in cell cycle‐metabolism interactions and can modulate the tumor immunological microenvironment through the interaction, mainly with CAFs and macrophage.

### 3.5 Suppression of HEC1 expression inhibited glioma cell proliferation through DNA replication and repair pathways

**FIGURE 5:**
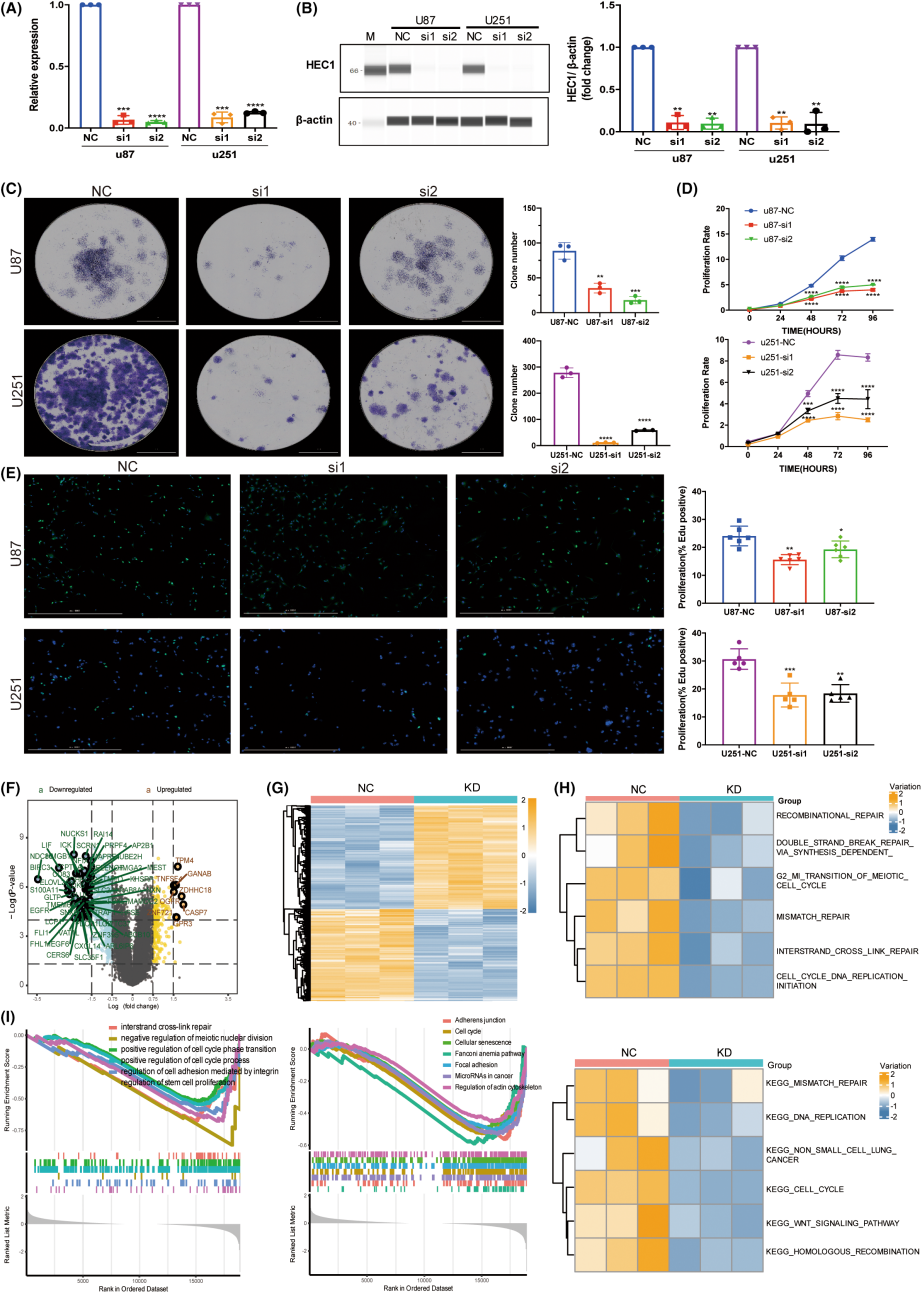
Suppression of HEC1 expression inhibited glioma cell proliferation via DNA replication and repair pathways. (A) The expression of HEC1 mRNA in U87 and U251 cell lines was examined by real‐time quantitative polymerase chain reaction. (B) HEC1 protein expression in U87 and U251 cell lines was examined by JESS. (C) HEC1 siRNA inhibits the ability to form clones in U87 and U251 cells. (D, E) HEC1 siRNA inhibits U87 and U251 cell proliferation detected by CCK8 assay (D) and EdU (E). (F, G) Detection of differential gene expression between control group and siRNA‐treated group in U251 cells. (H) Gene Ontology (GO, up) and Kyoto Encyclopedia of Genes and Genomes pathway (KEGG, down) enriched in two groups by Gene Set Variation Analysis (GSVA). (I) GO (left) and KEGG pathway (right) were enriched in two groups by Gene Set Enrichment Analysis (GSEA). *p < 0.5, **p < 0.01, ***p < 0.001, ****p < 0.0001, versus NC, *n* = 3–6.

Next, we attempted to verify the above findings on the regulatory role of HEC1 in glioma. The cells were transfected with siRNA to knockdown HEC1 expression (Figure 5A,B). After this, the number of clones formed in glioma cells was significantly reduced (Figure 5C). Furthermore, suppression of HEC1 suppressed glioma cells proliferation, as demonstrated by CCK8 and EdU assays (Figure 5D,E).

To understand the role of HEC1 in glioma better, HEC1‐knockdown U251 cells were subjected to RNA‐seq, which identified 9383 downregulated genes (Figure 5F,G). Enrichment analysis of all the differentially expressed genes revealed the association of high HEC1 expression with cellular processes, including cell cycle, division, DNA repair, cytoskeleton, and intercellular junctions (Figure 5H) with DNA repair, while cell cycle‐related pathways and biological processes were downregulated by HEC1 knockdown (Figure 5I). These results further support the role of HEC1 in cell cycle regulation and DNA repair in glioma.

### 3.6 HEC1 mediates macrophage migration and polarization

**FIGURE 6:**
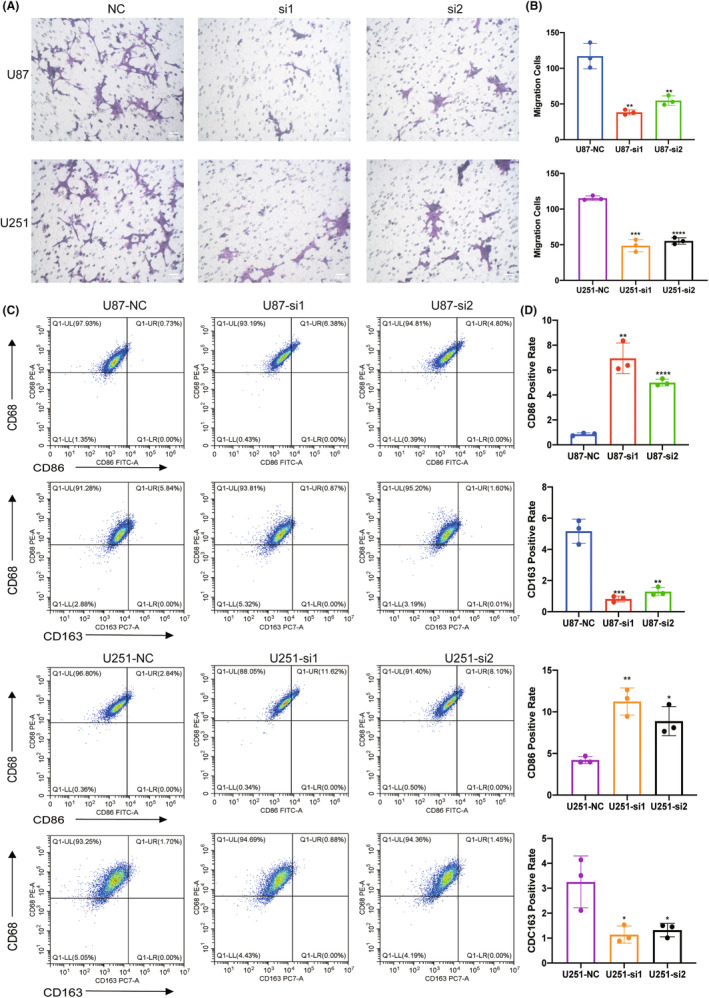
HEC1 derived from glioma cells mediated migration and polarization of HMC3 cells. (A) Representative images of transwell assay for migration of HMC3 cells in siRNA‐NC and siRNA‐HEC1 treated cells. (B) Statistical analysis of transwell assay. (C) Flow cytometry assay for the expression of CD68, CD86, and CD163 in siRNA‐NC and siRNA‐HEC1 groups. (D) Statistical analysis of flow cytometry data. *p < 0.5, **p < 0.01, ***p < 0.001, ****p < 0.0001, versus NC, *n* = 3.

**FIGURE 7:**
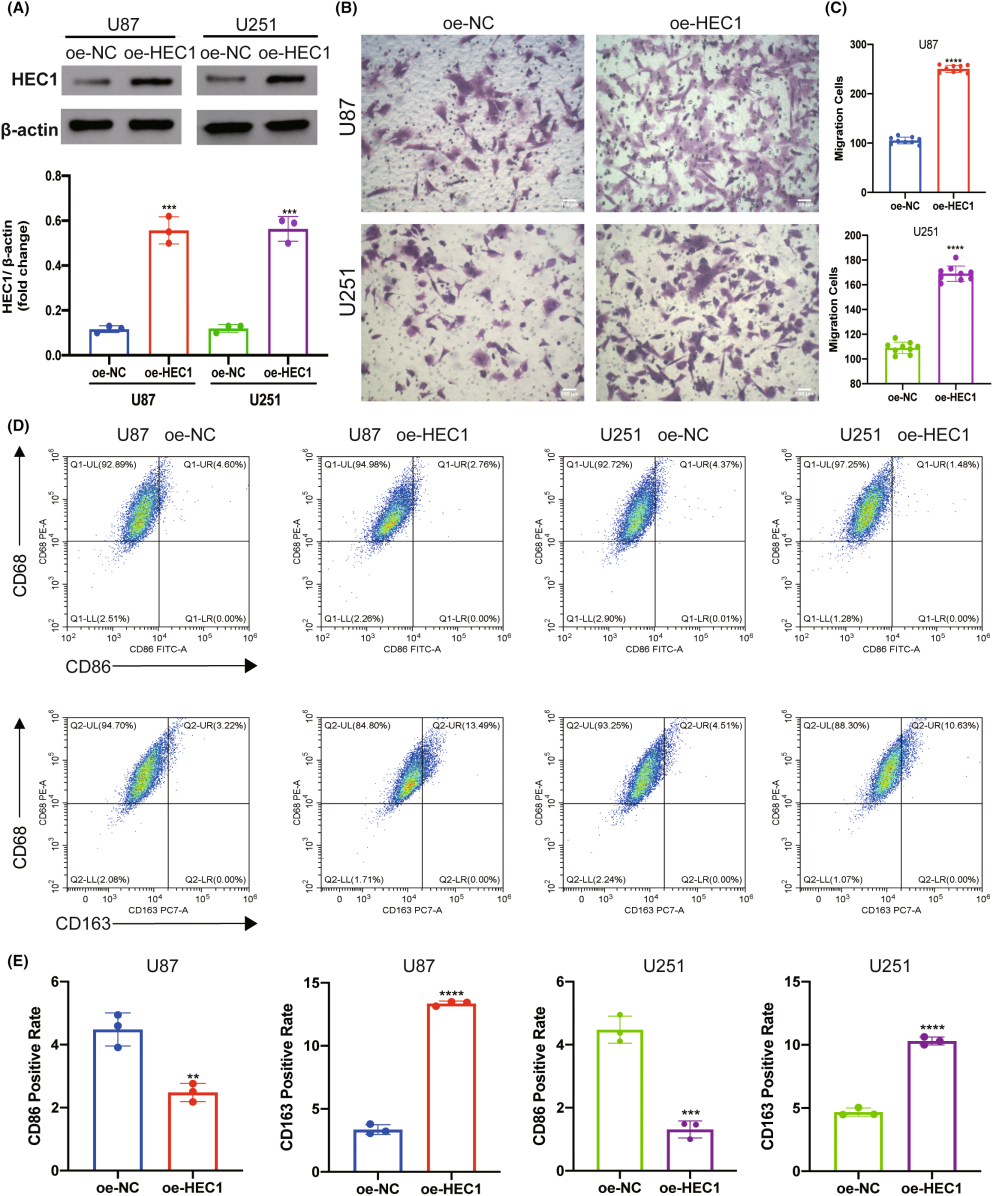
HEC1 derived from glioma cells mediated migration and polarization of HMC3 cells. (A) Overexpression of HEC1 protein in U87 and U251 cell lines was detected by western blot. (B) Representative images of transwell assay for migration of HMC3 cells in oe‐NC and oe‐HEC1 groups. (C) Statistical analysis of transwell assay. (D) Flow cytometry assay results of CD68, CD86, and CD163 expression in oe‐NC and oe‐HEC1 groups. (E) Statistical analysis of flow cytometry data. **p < 0.01, ***p < 0.001, ****p < 0.0001, versus oe‐NC, *n* = 3–8.

To confirm the interaction between HEC1‐mediated glioma cells and macrophages, the glioma cell line U87 and microglia cell line HMC3 were cocultured, the latter being a macrophage cell line in the central nervous system. U87 cells were transfected with HEC1 siRNA to suppress the expression of HEC1, as described previously. The transwell assay revealed that the migration of HMC3 cells in the HEC1 siRNA‐treated group was suppressed compared to that in the control siRNA‐treated group (Figure 6A,B). Flow cytometry assay showed that an increase in the number of CD68^+^CD86^+^ cells and the number of CD68^+^CD163^+^ cells was decreased (Figure 6C,D). Another glioma cell line U251 and HMC3 cells were also cocultured. U251 cells were treated with HEC1 siRNA or control siRNA and then cocultured with HMC3 cells. Like the U87 cells, treatment of U251 cells with HEC1 siRNA reduced the migration of HMC3 cells (Figure 6A,B). Compared with the control siRNA group, the number of CD68^+^CD163^+^ cells was reduced, while the number of CD68^+^CD86^+^ cells increased in the HEC1 siRNA‐treated group (Figure 6C,D). We then used plasmids to overexpress HEC1 (Figure 7A). The transwell assay was used to detect the migration of HMC3 cells after coculturing with U87. HMC3 cell migration in the HEC1 overexpressing plasmids‐treated group was promoted compared with the control group (Figure 7B,C). Flow cytometry assay further showed that the number of CD68^+^CD86^+^ cells decreased, while the number of CD68^+^CD163^+^ cells increased (Figure 7D,E). Like U87 cells, treatment of U251 cells with HEC1 overexpress plasmids resulted in enhanced migration of HMC3 cells (Figure 7B,C). Compared with the control group, the number of CD68^+^CD163^+^ cells increased, while the number of CD68^+^CD86^+^ cells decreased in the HEC1 overexpressing plasmids‐treated group (Figure 7D,E). The above results indicated the possible role of HEC1 in regulating the migration of microglia and promoting of conversion of microglia cells to the M2 phenotype.

**FIGURE 8:**
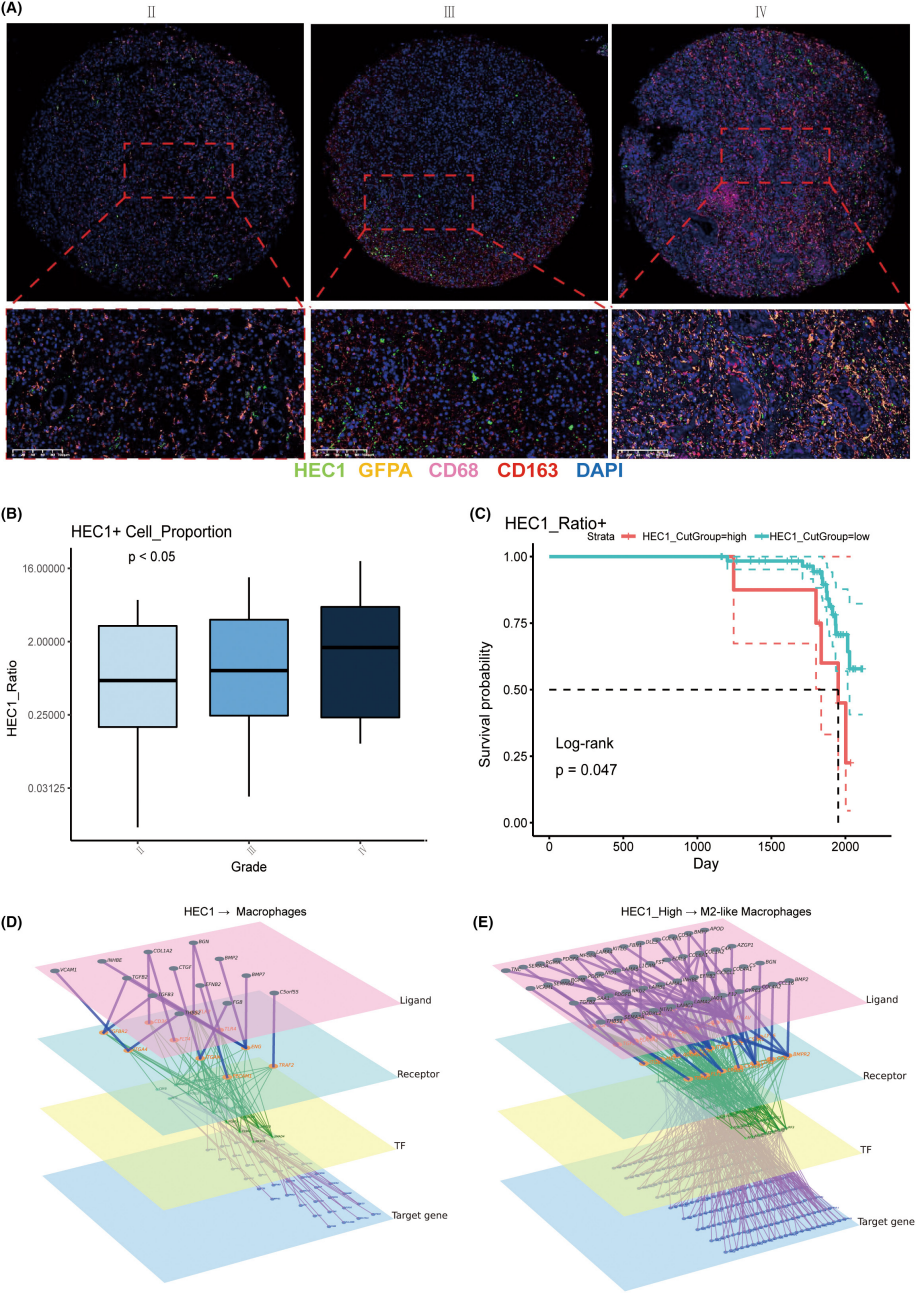
Interaction between macrophages and glioma cells. (A) Multiplex immunofluorescence staining of macrophage markers CD68, CD163, and glioma cell markers GFPA, and HEC1 in different pathological grades of gliomas. (B) The proportion of HEC1‐positive cell in different pathological grades of gliomas. (C) Kaplan–Meier analysis of overall survival of glioma patients based on high versus low expression of HEC1. (D) The multilayer network revealing the regulation of macrophage M2 marker genes by HEC1 through the downstream ligand, pathways, and transcription factors (TFs). (E) The multilayer network revealing the regulation of M2‐ike macrophage M2 marker gene by HEC1 through the downstream ligand, pathways, and TFs.

To further elucidate the biological importance of HEC1, we performed multiplex fluorescent immunofluorescence staining of macrophage marker CD68, CD163, along with glioma cell marker Glial fibrillary acidic protein (GFPA), across different grade of glioma (Figure 8A). Multiplex fluorescent immunofluorescence of human glioma tissue further showed positive correlation of HEC1 expression in glioma with the infiltration of M2 macrophages (CD68^+^CD163^+^) (Figure 8A), the malignant degrees of gliomas (Figure 8B), and a lower survival probability of patients with high HEC1 expression (Figure 8C). We used the multilayered network (scRNA‐seq‐based tools) for cell–cell communication to analyze HEC1‐mediated cell–cell interactions. The multilayer network revealed that HEC1 may related to many ligands in macrophage related to M2 polarization (e.g., BMP7, THBS2, and TGFB2), and activates multiple transcription factors (e.g., CEBP, STAT1, SMAD4, and SAMD3). Furthermore, the downstream target genes involve many marker genes related to macrophage polarization (e.g., IRF8, IL‐2, CD86, PLD1, SMAD7, TLR10, and IL10) (Figure 8D,E).

## 4 DISCUSSION

HEC1 is one of the key elements of the kinetochore and can strongly support the formation of a heterotetrameric protein complex with a critical role in cell mitosis.^31^ Aberrant expression of HEC1 is associated with chromosomal abnormalities potentially leading to tumorigenesis.^14^, ^17^ Studies have shown that high HEC1 expression is related to tumor grade, and HEC1 is involved in the regulation of cell cycle, apoptosis, proliferation, and metastasis in human pancreatic cancer,^11^ HCC,^14^ colon cancer,^17^ and prostate cancer.^32^ Meanwhile the potential functions of HEC1 in glioma have been rarely reported. A previous study found increased HEC1 expression in GBM and positively correlated to the degree of malignancy, and HEC1 could accelerate the proliferation and invasion ability of GBM cells,^21^ but its specific biological function and mechanisms in glioma yet to be elucidated. Here, we again found that HEC1 is highly expressed in glioma, and its high expression was associated with malignant clinical features and worse prognosis.

To understand the role of HEC1 better, Bulk RNA‐Seq and scRNA‐Seq data were screened and enrichment analysis was also performed. HEC1 was found positively associated with the glioma cell cycle, DNA repair and TME. HEC1 is the major component of the kinetochore complex and participates in the mitotic chromosome division.^33^ Similarly, we found that the tumor cells in glioma samples with high HEC1 expression had higher rate of DNA replication, metabolism‐related biological process, and DNA‐related biological processes, including E2F target, G2M checkpoint, and DNA repair. The influence of HEC1 on cell cycle and DNA‐related biological processes was further confirmed by RNA‐seq of glioma cells harboring HEC1 knockdown. Among the genes that possibly regulate HEC1, E2F8 is a member of the E2F family of transcription factors that regulate cell differentiation, proliferation, cell cycle, apoptosis, and DNA repair.^34^ Recent studies reported overexpression of E2F8 in various cancers,^35^, ^36^, ^37^ suggesting that E2F8 plays an important role in tumorigenesis. The organization of the DNA‐binding domains of E2F8 is similar to that of E2F7.^38^ Several target genes bearing E2F7‐binding sites can be directly repressed by E2F7, while most of these targets are involved in regulating DNA activity.^39^ E2F8^40^ and E2F7^39^ can regulate the cell cycle; reports from literature suggest similar function for E2F8 and E2F7 in regulation of cell cycle and DNA activity. In this study, we too observed remarkably high similarity of E2F8 and HEC1 in tumor cell distribution. A comparison of the E2F8 Chromatin Immunoprecipitation sequencing (ChIP‐seq) data in the Jaspar database with the HEC1 promoter sequence revealed a possible binding. Therefore, E2F8 possibly affects HEC1‐mediated DNA replication and repair in tumor cells through the regulation of HEC1 expression.

TME comprises cellular components and a noncellular matrix, and is a key factor in tumor occurrence and development.^4^ Infiltrating cells in the TME of glioma include immune cells, stromal cells, and other non‐tumor glial cells.^4^, ^41^ Some previous studies have linked the NDC80 kinetochore complex component HEC1 to immune cell infiltration and tumor‐specific epitopes.^42^, ^43^, ^44^ HEC1 is one of the major neighboring genes of the Cell Division Cycle Associated (CDCA) family genes. CDCA family genes lead to poor outcomes and correlate positively with the infiltration of macrophage, dendritic cells, and B cells in HCC.^19^, ^45^ However, to our knowledge, no previous research has ever focused on the interaction between HEC1 and the TME. In the current study, we observed that macrophage and CAFs are enriched in the glioma samples overexpressing HEC1. Furthermore, most immune checkpoints are highly expressed in glioma samples with high HEC1 expression. Our findings showed for the first time that HEC1 can interact with the TME in glioma.

Macrophage and CAFs are two important types of cells in glioma TME. Macrophage is a type of innate immune cells that form a heterogeneous and plastic part of the glioma TME, especially GBM.^41^ Macrophage can act as a double‐edged sword, as it can promote tumor progression and drug resistance by supplying malignant cells with nutrition and energy. On the other hand, macrophage mediates antineoplastic effects through phagocytic and oxidative functions.^46^, ^47^ While for CAFs, they are rich sources of various secreted factors such as proinflammatory cytokines, inflammatory ligands, growth factors, and ECM proteins, which can trigger proliferation, chronic inflammation, immune defense, and render resistance to therapy.^48^, ^49^, ^50^ Our results showed an increase in the number of infiltrating macrophages and CAFs in glioma samples with high HEC1 expression. Compared with the low HEC1 expression group, the high HEC1 expression group interacted more with macrophages and CAFs. Our findings suggested that high expression of HEC1 may increase the tumor cell interaction with macrophage and CAFs, and shape TME in glioma. Macrophages are roughly classified into two states, including M1 and M2. The M1 subtype is associated with pro‐inflammation, while the M2 subtype is associated with anti‐inflammation.^51^ However, this classification is probably too simplistic. Microglia are also divided into an M1‐like and an M2‐like subtypes. In glioma, M1‐like macrophages are involved in the inflammatory response, pathogen clearance, and anti‐tumor immunity, whereas M2‐like macrophages participate in anti‐inflammatory response, wound healing, and pro‐tumor properties.^52^ In the current study, HEC1 of glioma cells mediated the migration and polarization of microglia in the glioma TME. When HEC1 expression was disrupted in glioma cells, this could lead to inhibition of microglial migration. The enhanced expression of CD86 and reduced expression of CD163 in microglia suggested that HEC1‐derived glioma cells could promote M2 polarization. The multilayered network (scRNA‐seq‐based tools) was used to analyze HEC1‐mediated cell–cell interactions. The multilayer network revealed that HEC1 may related to many ligands in macrophage related to M2 polarization (e.g., BMP7, THBS2, and TGFB2), and activates multiple transcription factors (e.g., CEBP, STAT1, SMAD4, and SAMD3). Furthermore, the downstream target genes involve many marker genes related to macrophage polarization (e.g., IRF8, IL‐2, CD86, PLD1, SMAD7, TLR10, and IL10). M2 macrophages contribute to angiogenesis,^53^ ECM remodeling,^54^ immunosuppression,^55^ and tumor progression.^56^ Hence, HEC1 may promote the development of glioma by inducing M2 polarization of macrophages via receptor ligands.

Immune checkpoint refers to programmed death receptors and their ligands, which are specific molecule that can modulate the T cells interaction with antigen‐presenting cells and inhibit the function of T cells.^57^ Immune checkpoint signaling pathway dysregulation is an important mechanism for immune escape of tumor cells.^58^ Some immune checkpoint inhibitors, including antibodies and small molecules enhance the anti‐tumor immune response, are universally effective against a wide range of cancers and have led to significant clinical advances.^59^ The *PDCDL1* gene encodes a protein named PD‐L1, also known as CD274 and B7‐H1.^60^ The activity of PD‐L1 depends on its binding to the programmed cell death 1 (PD‐1) receptor, encoded by the *PDCD1* gene, on the target immune cells.^61^ The PD‐1/PD‐L1 axis facilitates glioma cell invasion in the brain. PD‐L1 secreted from glioma cells binds to the PD‐1 receptor in microglia, resulting in a negative regulation of the immune responses.^62^ We found a strong expression of most immune checkpoints, including *PDCDL1* and *PDCD1*, in tumor cells of glioma samples overexpressing HEC1. This finding suggests possible role of HEC1 in the formation or maintenance of inhibitory immune microenvironment in glioma.

This study also had some limitations. According to the study by Li et al.,^63^ the glioma cell lines are a poor representative of primary human glioma. First, they found that U251, U373, and SNB‐19 belong to the same cell lines. Comparison of the copy number aberration (CNA) profiles in A172, Hs683, T98G, U251, and U87 showed significant difference of U87 from the other cell lines. Both primary human glioma and the cell lines share upregulated typical oncogenic signals such as *p53* and *Wnt* signaling pathways. Nevertheless, differences in gene expression in many areas were observed, such as G protein‐coupled receptor signaling. In contrast, in a functional study, genes related to cell cycle and oxidative phosphorylation are preferred. However, the characteristics of primary human glioma cells were difficult to retain in vitro culture system. Therefore, U251 and U87 cell lines used in our study were established by previous reported studies.^64^, ^65^ Fortunately, HEC1 was associated with upregulated cell cycle in both primary human glioma cells and the glioma cell lines; therefore, the study was still credible. The experiments with primary cells need to be further investigated.

## 5 CONCLUSIONS

High expression level of HEC1 was correlated with malignant clinical features, immune cell infiltration, immune checkpoints, and poor survival. HEC1 promotes development of glioma through the regulation of proliferation, cell cycle, DNA repair, and TME formation, possibly through transcriptional activation of E2F8. Moreover, tumor cells exhibiting HEC1 expression could shape the TME by interacting with macrophage and CAFs. Glioma cell‐derived HEC1 mediated macrophage migration and polarization. This is the first study to show positive association of HEC1 with tumor immunity in glioma. HEC1 may be a candidate prognostic marker and could be a potential target in the immunotherapy of glioma.

## AUTHOR CONTRIBUTIONS

Weijie Ye performed the experiments, analyzed data, and wrote the original manuscript. Xisong Liang performed the experiments, analyzed data, and revised the manuscript. Ge Chen and Qiao Chen performed the experiments and revised the manuscript. Hao Zhang, Nan Zhang, and Yuanfei Huang analyzed data and revised the manuscript. Quan Cheng and Xiaoping Chen designed the study, acquired funding, provided supervision and conceptualization, and revised the manuscript. All authors have read and agreed to the published version of the manuscript.

## FUNDING INFORMATION

This study was supported by the Hunan Provincial Natural Science Foundation of China (NO. 2022JJ20095), China Postdoctoral Science Foundation (NO. 2018M633002), Hunan Youth Science and Technology Talent Project (NO. 2023RC3074), and Fundamental Research Funds for the Central Universities of Central South University (2020zzts274).

## CONFLICT OF INTEREST STATEMENT

The authors declare no conflicts of interest.

## Supporting information


FigureS1

FigureS2

FigureS3

FigureS4

FigureS5

FigureS6

DataS1

## Data Availability

Availability of data and materials: Data used in this work can be acquired from the public database and so on. Other datasets generated in the current study could be available by contacting the corresponding author.
