# Supplementary figures and images for: NDC80/HEC1 promotes macrophage polarization and predicts glioma prognosis via single‐cell RNA‐seq and in vitro experiment
Source: CNS Neurosci Ther. 2024 Jul 17;30(7):e14850. doi: 10.1111/cns.14850 (PMC11255415; doi:10.1111/cns.14850)

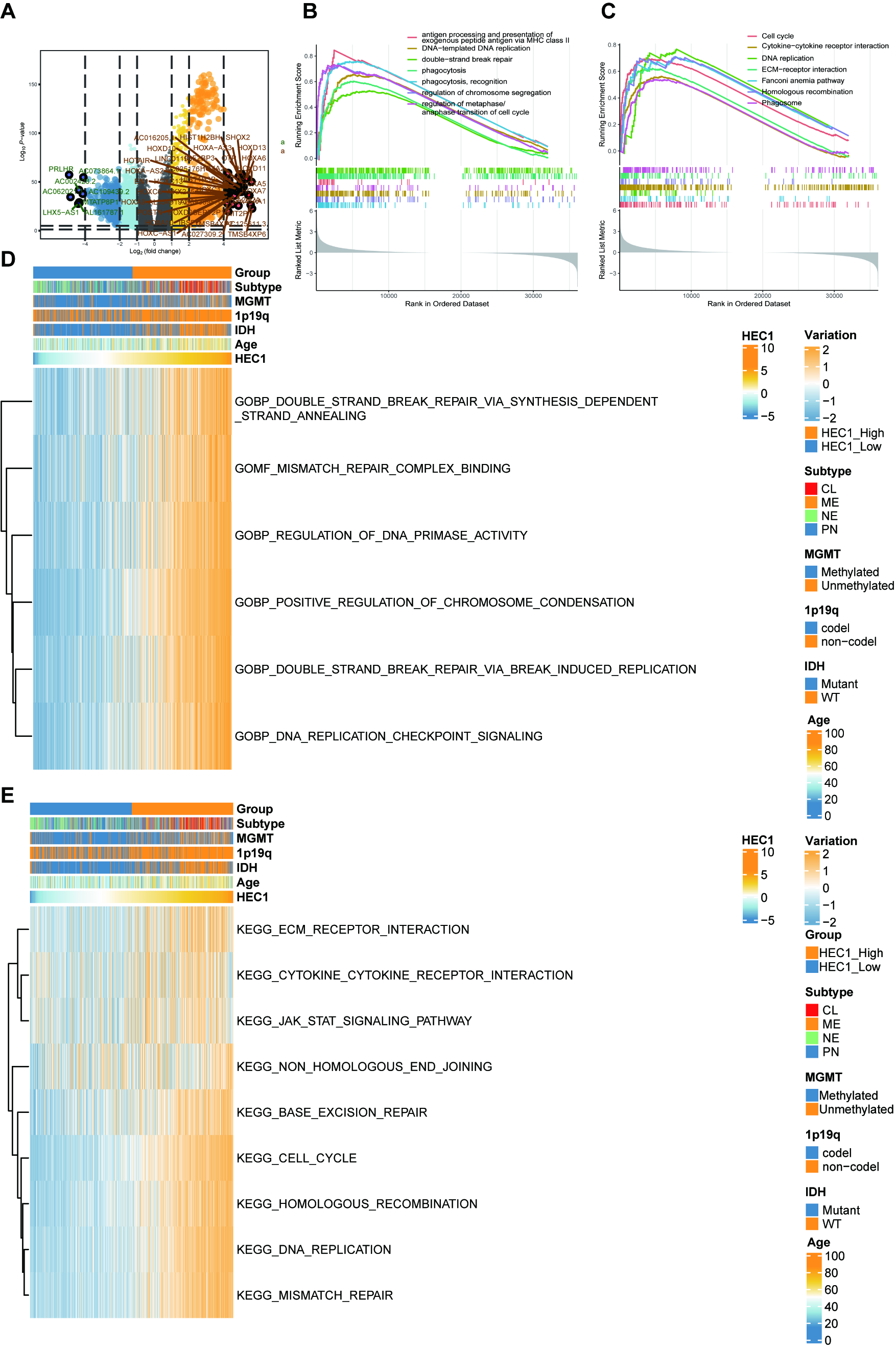

Supplement: Supplementary file 1 — FigureS1 [file CNS-30-e14850-s005.tif]

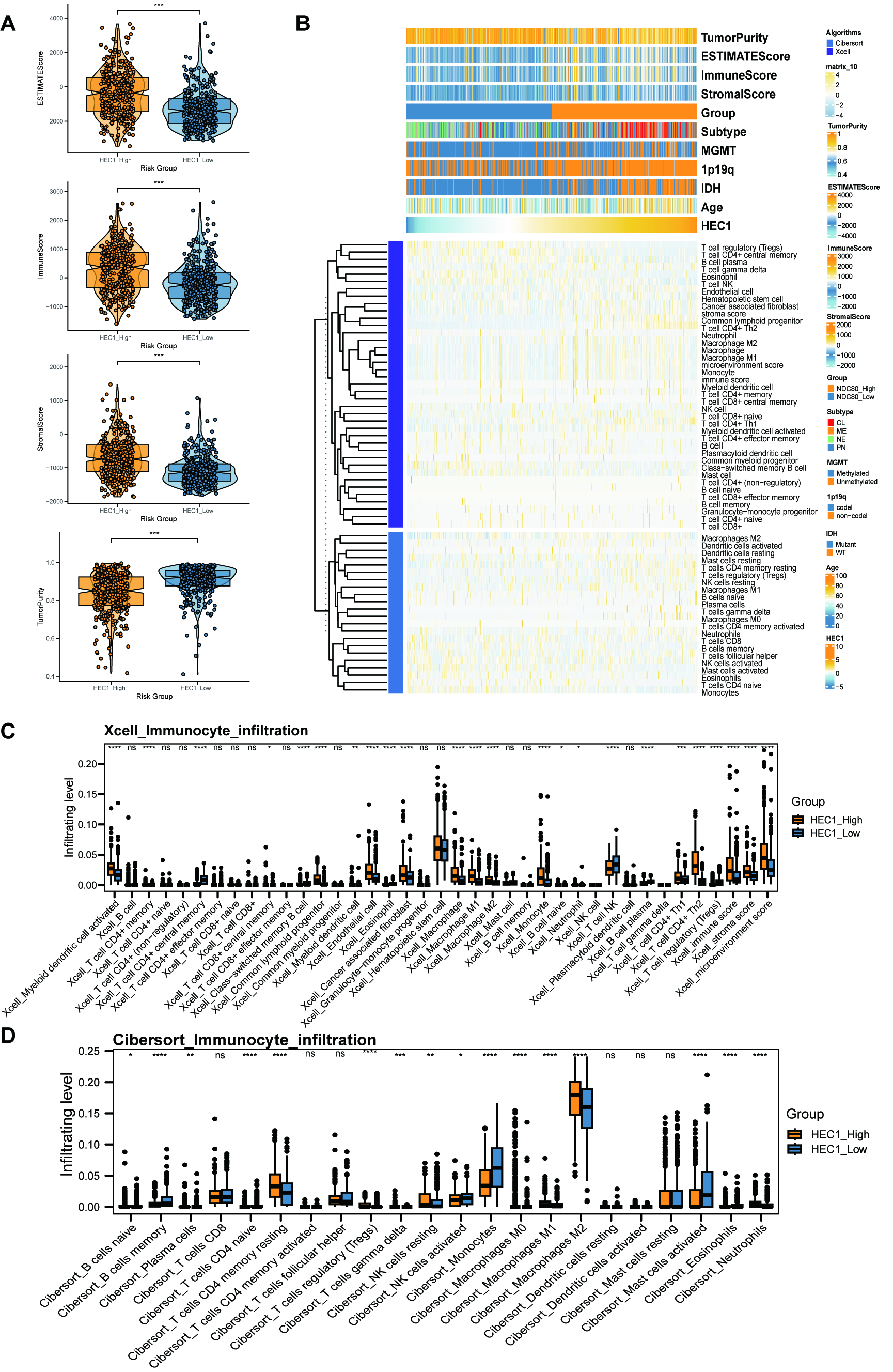

Supplement: Supplementary file 2 — FigureS2 [file CNS-30-e14850-s006.tif]

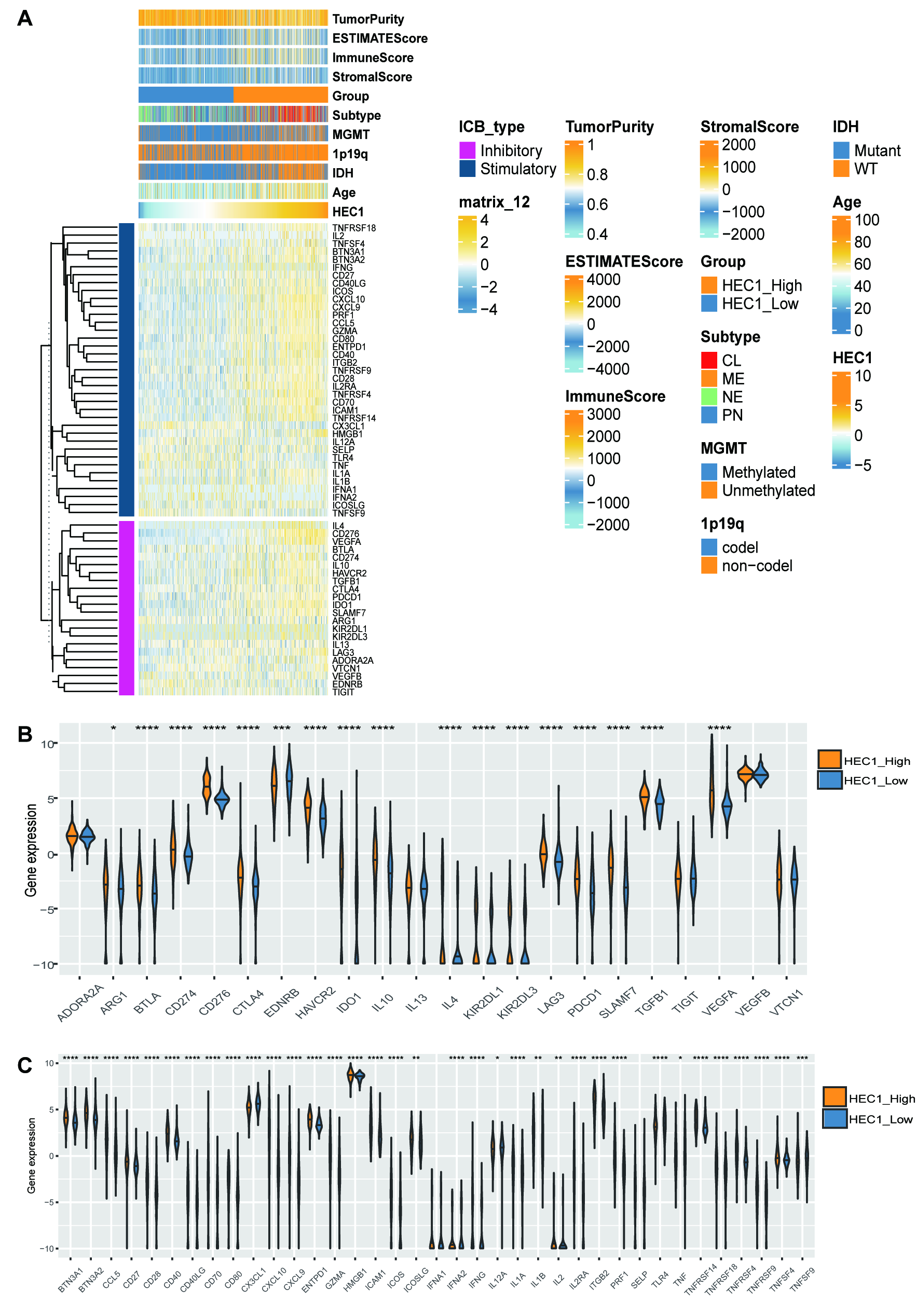

Supplement: Supplementary file 3 — FigureS3 [file CNS-30-e14850-s003.tif]

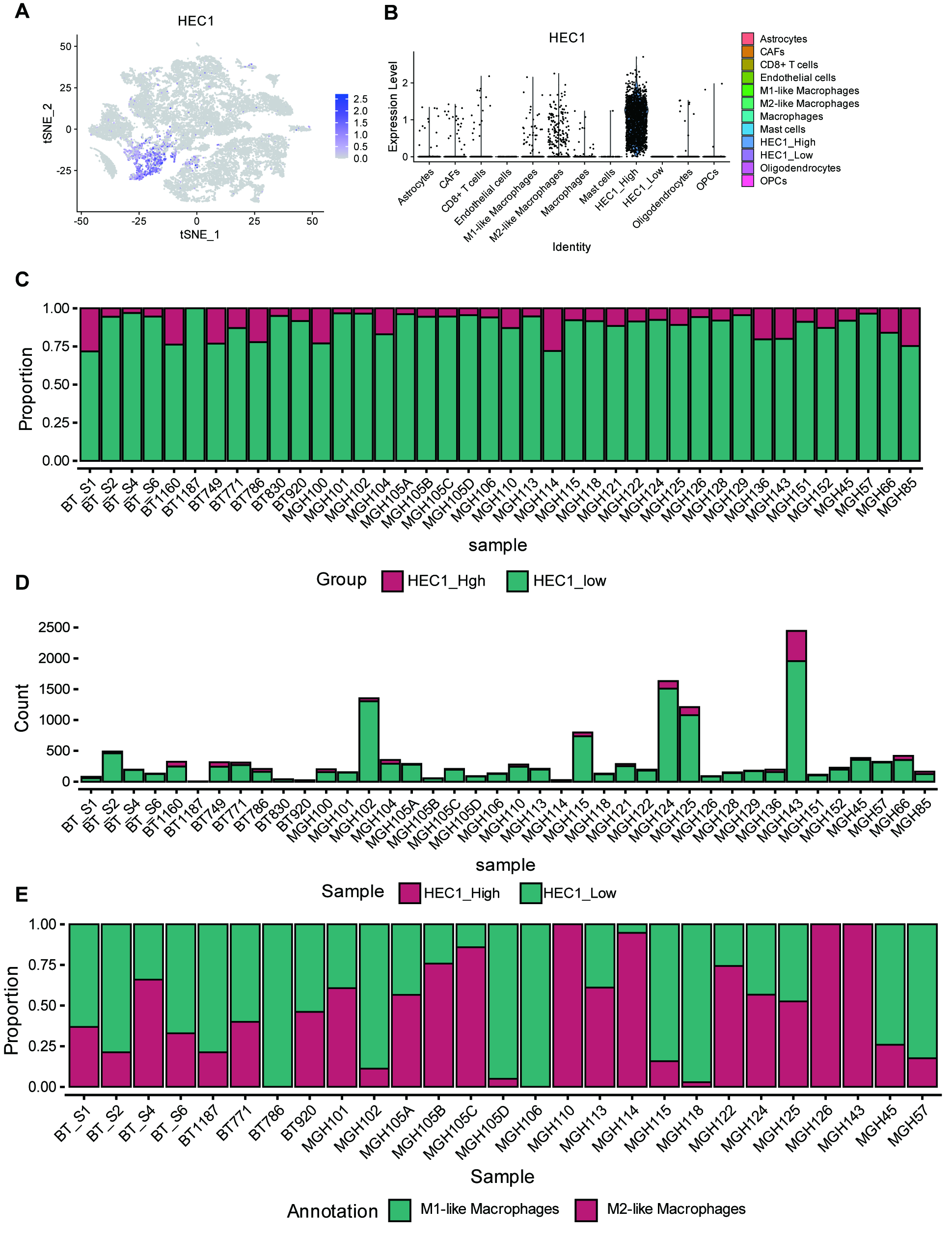

Supplement: Supplementary file 4 — FigureS4 [file CNS-30-e14850-s004.tif]

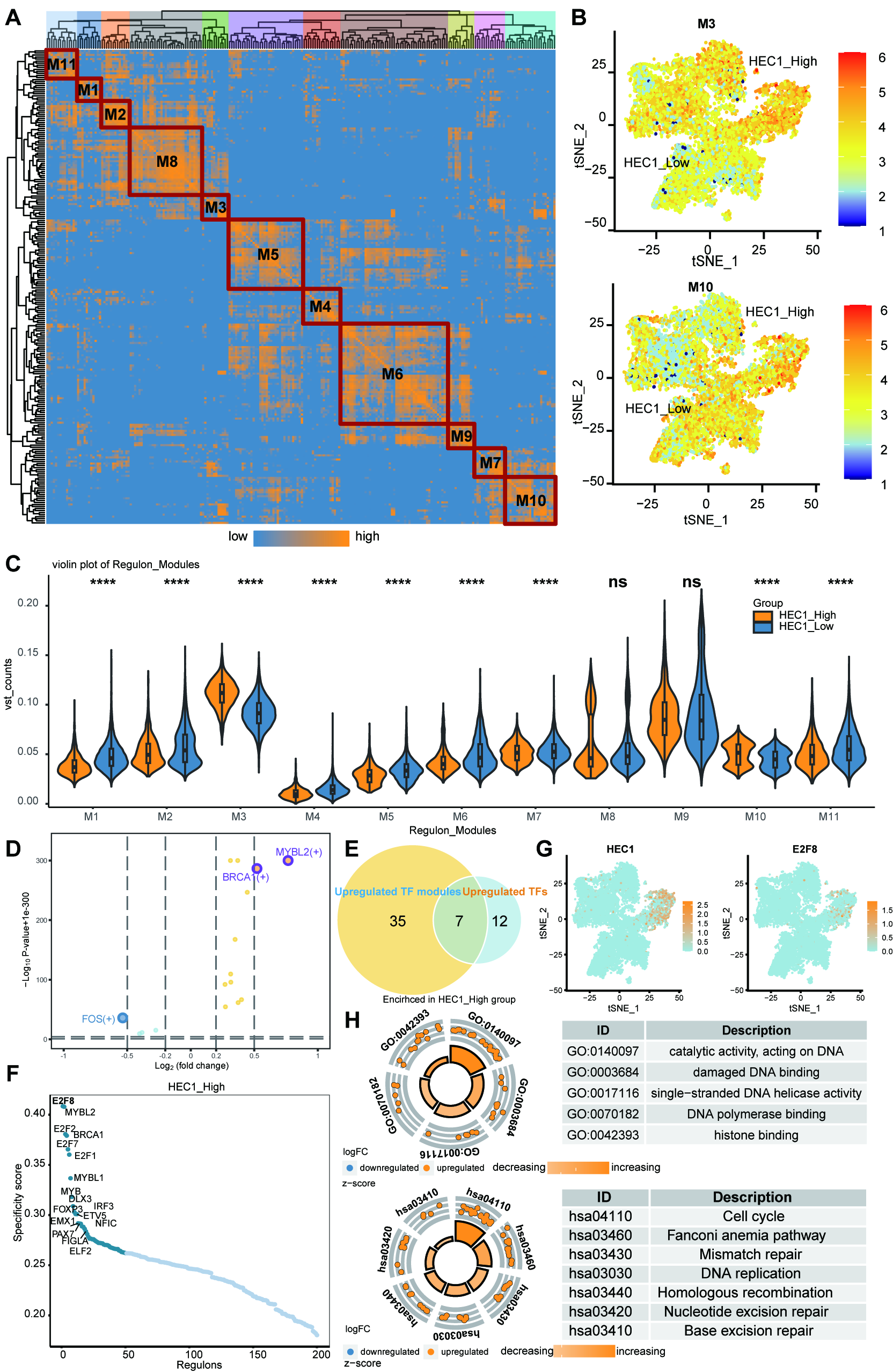

Supplement: Supplementary file 5 — FigureS5 [file CNS-30-e14850-s002.tif]

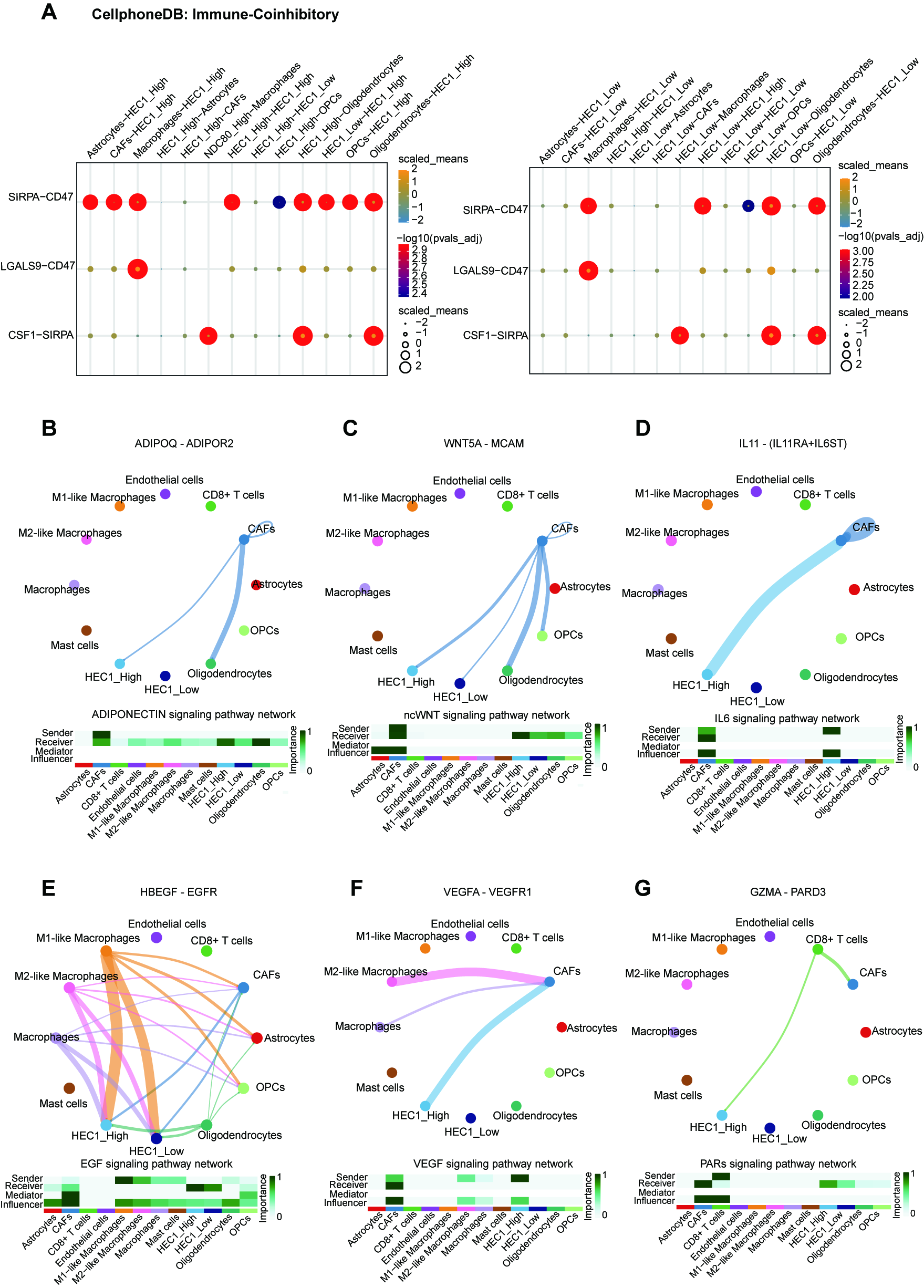

Supplement: Supplementary file 6 — FigureS6 [file CNS-30-e14850-s007.tif]

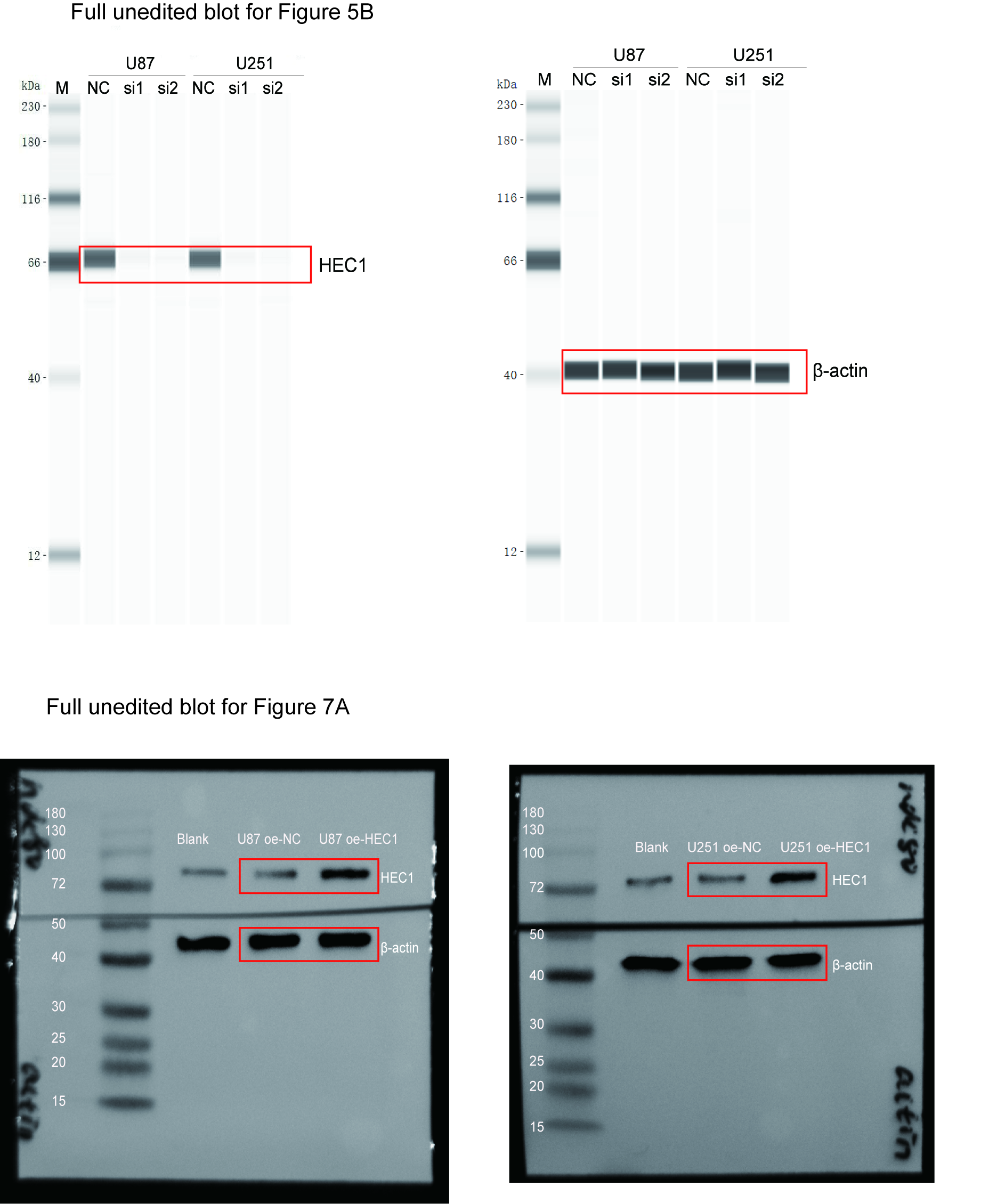

Supplement: Supplementary file 7 — DataS1 [file CNS-30-e14850-s001.tif]
